# In Vitro Determination of Antimicrobial, Antioxidant and Antiviral Properties of Greek Plant Extracts

**DOI:** 10.3390/microorganisms13010177

**Published:** 2025-01-16

**Authors:** Aliki Tsakni, Eirini Kyriakopoulou, Sophia Letsiou, Panagiotis Halvatsiotis, Haralambos Rigopoulos, Niki Vassilaki, Dimitra Houhoula

**Affiliations:** 1 Department of Food Science and Technology, Faculty of Food Sciences, University of West Attica, 12243 Athens, Greece; aliki_tsak@yahoo.gr (A.T.); sletsiou@gmail.com (S.L.); h.rigopoulos@progressinc.gr (H.R.); 2 Laboratory of Molecular Virology, Hellenic Pasteur Institute, 11521 Athens, Greece; eirhnh63@gmail.com (E.K.); nikiv@pasteur.gr (N.V.); 3 Department of Biomedical Science, University of West Attica, 12243 Athens, Greece; 4 2nd Propaedeutic Department of Internal Medicine, Medical School, National and Kapodistrian University of Athens, “ATTIKON” University Hospital, 12461 Chaidari, Greece; pahalv@gmail.com

**Keywords:** natural extracts, antioxidant activity, antimicrobial properties, cytotoxicity, dengue virus

## Abstract

The medicinal potential of plant extracts, especially their antimicrobial, antioxidant, antiviral and cytotoxic properties, has gained significant attention in recent years. This study examined the in vitro bioactivities of several selected Greek medicinal plants, like *Eucalyptus globulus* L., *Thymus vulgaris* L., *Salvia rosmarinus* L. and *Ocimum basilicum* L., are well-known for their traditional therapeutic use. Minimum inhibitory concentration (MIC) assays were used to evaluate the antimicrobial activity of the extracts against pathogenic bacteria. The antioxidant activity was carried out using the DPPH method, while the cytotoxicity of the plants was determined using the Alamar Blue method. In addition, the antiviral efficacy of the samples was tested against DENV in different cell lines. The majority of medicinal herbs demonstrated significant antimicrobial action (MIC = 30–3000 μg∙mL^−1^). The extracts showed great antioxidant activity, while the *Salvia rosmarinus* L. extract turned out to be the most effective (IC_50_ = 12.89 ± 0.11 μg∙mL^−1^). In contrast, the extract of *Eucalyptus globulus* L. had the lowest antioxidant action (IC_50_ = 71.02 ± 0.42 μg∙mL^−1^). The results of the Alamar Blue method were presented with CC_50_ values, and it was shown that *Eucalyptus globulus* L. extract exhibited the highest cytotoxicity (CC_50_ = 5.94% *v*/*v* ± 0.04). Similarly, the results of the antiviral potential of extracts were expressed as EC_50_ values, and *Eucalyptus globulus* L. was characterized as the most effective sample against dengue virus infection, with EC_50_ values estimated at 2.37% *v*/*v* ± 0.6 (HuhD-2 cells infected with DENV-2) and 0.36% *v*/*v* ± 0.004 (Huh7.5 cells infected with DVR2A). These findings provide a foundation for further studies in order to combat infectious diseases and promote human health.

## 1. Introduction

The Mediterranean area has a diverse range of medicinal and aromatic plants that thrive in various environments. Greece’s geographical location, geomorphology, flora and interaction of biotic and non-biotic factors make it a region with high plant diversity and endemism, having an impact on medicinal and aromatic plants [1]. The Greek medicinal plants are known for their important properties as well as their uses in different sectors in human life since ancient times [1].

Specifically, the *Eucalyptus globulus* leaves are a rich source of terpenoids, tannins, flavonoids, flavonoid glycosides, ellagic acid and derivatives, galloyl glucose derivatives and ellagitannins. In addition, *Eucalyptus globulus* Labill is reported to possess antioxidant, antiradical, antidiabetic, insecticidal and antibacterial activity [2,3]. The thyme (*Thymus vulgaris* L.), a member of the Lamiaceae family, contains a high concentration of essential oils, phenolic compounds and flavonoids, which lead to powerful antioxidant, antimicrobial and anti-inflammatory properties [4]. In addition, thymol and carvacrol, the primary components of thyme, are especially active in counteracting induced oxidative stress. These bioactive compounds have been reported to improve immune function, reduce inflammation and possess antimicrobial properties against a wide range of pathogens [5].

*Salvia rosmarinus* (L.) is an evergreen shrub found mainly in the Mediterranean region, known for its high resistance to summer drought [6,7,8]. Thus, *Salvia rosmarinus* is prone to oxidative damage in extreme drought circumstances, particularly when its antioxidant defences are insufficient to combat the subsequent oxidative stress, resulting in photosynthetic damage [9,10,11,12]. In addition, *Ocimum basilicum* L. belongs to the members of the Lamiaceae family of aromatic plants and is among the medicinal plants used for this purpose [13]. A wide range of diseases, such as diabetes, infectious diseases, hemorrhoids, cardiovascular diseases, fevers, nerve pains and inflammation, have historically been treated using the herbal extract of *Ocimum basilicum* L. [14]. Essential components of this plant, like chlorogenic acid, rosmarinic acid and apigenin, are responsible for its strong capacity to scavenge free radicals [15]. Additionally, it has been observed that *Ocimum basilicum* L. has antimicrobial action against Gram-positive and Gram-negative bacteria, cytotoxic properties [16], chemopreventive and anticancer activities [17], antihypertensive and cardioprotective activities [18]. Bioactive compounds, such as linalool and eugenol, also explain the antidiabetic effects [19].

On the other hand, plant extracts may possess antiviral properties, offering cost-effective and less harmful alternatives to synthetic drugs [20]. Worldwide, flaviviridae viral infections—such as yellow fever (YFV), dengue (DENV), and hepatitis C—pose serious threats to public health. While YFV is still endemic in many areas, mosquito-borne DENV (Flavivirus genus) infects an estimated 400 million people annually in more than 100 countries, despite successful immunization [21]. In addition, it has been reported that disorders of the central and visceral nervous systems can be caused by DENV and YFV [22]. The clinical manifestations of DENV infection vary widely, ranging from mild fever to severe conditions like dengue haemorrhagic fever and shock syndrome [23]. The existing dengue vaccine has shown only limited efficacy, and no antiviral treatment has been approved to date. Climate change is facilitating the spread of mosquito vectors and DENV, even in countries that have not experienced dengue epidemics in recent years. This includes the establishment of DENV-transmitting mosquito species in Greece, raising concerns about the country’s potential vulnerability to future outbreaks [24].

Plant-based alternatives provide a vital chance to fight viral infections and antibiotic resistance in a world where conventional therapies are becoming less and less successful [25,26]. They provide a variety of mechanisms, sustainability and drug discovery opportunities, making them a crucial part of the worldwide approach to controlling infectious illnesses and reducing dependency on synthetic drugs [27]. Herbal products could also be crucial in filling healthcare gaps, particularly in underprivileged areas where access to traditional therapies is restricted [28].

Currently, there is a tendency in the scientific community to explore natural extracts in an attempt to be used in various sectors of human health, leading to innovative drug-related products such as pharmaceuticals, nutraceuticals or cosmeceuticals. Even though there are many natural extracts with important properties for human life, only a few of them have been sufficiently scrutinized, especially for their antiviral properties. Thus, in this study, we investigate the in vitro antimicrobial, antioxidant and antiviral properties of *Eucalyptus globulus* L., *Thymus vulgaris* L., *Salvia rosmarinus* L. and *Ocimum basilicum* L. in an attempt to unravel their essential properties that may combat infectious diseases and promote human health. The novelty of this research focuses on the study of plants whose antiviral and cytotoxic properties have not been demonstrated before. Another innovation of this survey is that it examines a combination of multiple biological processes, offering a more comprehensive understanding of the plant’s potential medicinal benefits and therapeutic uses. These investigations are promising for new drug development, consisting of natural compounds that can treat infections, oxidative stress or even cancer.

## 2. Materials and Methods

### 2.1. Chemicals

Ethanol (purity ≥ 99.9%) and methanol (analytical grade) were supplied by Merck (Darmstadt, Germany). DPPH (2,2-diphenyl-1-picrylhydrazyl), Folin–Ciocalteu reagent and sodium carbonate (Na_2_CO_3_) were acquired from DR EHRENSTORFER GmbH (Augsburg, Germany). Alamar Blue reagent was purchased from Sigma-Aldrich (St. Louis, MO, USA).

### 2.2. Preparation and Extraction of the Samples

All plant materials, *Eucalyptus globulus* L., *Thymus vulgaris* L., *Salvia rosmarinus* L. and *Ocimum basilicum* L., were collected from Crete, an island that belongs to Greece, in June 2022. Their dried leaves were ground in a mechanical blender. The powdered material of each plant (10 g) was macerated in an Erlenmeyer flask (100 mL) [29]. The solvent used was 90% distilled water and 10% ethanol. This solvent system is considered to be safe for developing new products, cost-effective, eco-friendly and efficient at preserving bioactive components. The mixture was kept for 2 weeks in the dark and at room temperature with frequent agitation. After filtering, the natural extract was centrifuged at 8000× *g* force for 15 min, and the supernatant was collected. The rotary evaporator was operated at 50 °C for 2 h to remove the solvent [30]. The solid residue was dissolved in the suitable solvent and stored at 2 °C for further experiments. For example, the assessment of antioxidant capacity was conducted using methanol, while water was used in order to evaluate the antimicrobial and cytotoxic effects of natural extracts.

Plant extracts need to be stored properly in order to remain stable and potent. The extracts’ stability is influenced by a number of variables, including temperature, light, air exposure and the type of solvent used [31]. The plant extracts were stored at 2 °C because cold storage slows the deterioration of heat-sensitive active ingredients. In addition, the extracts were stored in darkness, as exposure to light can weaken the efficacy of phytochemical substances and induce oxidative stress. Furthermore, the extracts were kept in airtight containers to avoid oxidation, which can degrade bioactive compounds like polyphenols.

### 2.3. Antioxidant Properties

The DPPH (1,1-diphenyl-2-picrylhydrazyl) method was mainly developed to assess the antioxidant capacity of plant or food extracts. The principle of this method is the donation of electrons by antioxidants to neutralize the DPPH radical. A sign of antioxidant activity is the change in colour of the solution from purple to yellow [32]. A 6 × 10^−5^ M radical stock solution in methanol was prepared prior to the analysis. Plant extracts (100 μL) in different concentrations (8.44, 16.88, 33.75, 67.50, 135 μg·mL^−1^) were combined with 3400 μL DPPH solution [33]. The mixtures were incubated for 45 min in the dark at room temperature. The decrease in absorbance (Asample) was monitored at 517 nm with a VIS spectrophotometer (Thermo Spectronic Helios Epsilon, Waltham, MA, USA). The same procedure was used to prepare a control sample, which contained 3400 μL DPPH solution and 100 μL methanol. The absorbance of the control was measured at 517 nm (Acontrol) [34]. All the measurements were performed three times. The following equation was used to determine the radical scavenging activity:

(1) $$\%\;Radical\;scavenging\;activity=\frac{Acontrol-Asample\;}{Acontrol}\times 100\%\;$$

Several approaches may be used to overcome potential measurement biases and guarantee the reproducibility of antioxidant activity measurements across replicates. First, multiple replicates of each sample were performed to ensure the antioxidant activity measurements were accurate. Consequently, experimental errors and consistent results were obtained. In addition, every step was meticulously carried out in accordance with the protocol described above for sample preparation, solvent selection and analysis to minimize measurement bias. For instance, the plant extract’s concentration was the same in each replicate. It was also crucial to use the same equipment (spectrophotometer, cuvettes) for every measurement in order to ensure accuracy and avoid discrepancies in results. The spectrophotometer was also calibrated consistently to provide repeatable results. Finally, the DPPH reagent used was prepared daily and properly stored to prevent degradation, which could lead to inaccurate measurements. These approaches ensure repeatability and reduce measurement biases.

### 2.4. Total Phenolic Content—Folin–Ciocalteu Method

The phenolic constituents included in the plant extracts have redox properties, which give them their antioxidant activities. The Folin–Ciocalteu method was used to determine the total phenolic content of the samples [35]. Plant extracts (200 μL) (8.44–135 μg·mL^−1^) were combined with 1 mL Folin–Ciocalteu reagent (10% *v*/*v*) and 800 μL Na_2_CO_3_ (7.5% *w*/*v* in deionized water). The mixture was incubated for 1 h in the dark at room temperature, and its absorbance was measured at 765 nm with Thermo Spectronic Helios Epsilon (USA). Quantification was carried out using the gallic acid standard curve. The standard reference curve was constructed using five different concentrations of gallic acid (12.5, 25.0, 50.0, 100.0, 200.0 μg∙mL^−1^). The equation representing the curve was as follows:

(2) $$y=0.0118x\;–\;0.0819\left(R^{2}=0.9953,\;p<0.05\right)\;$$

### 2.5. Determination of Antimicrobial Activity

#### 2.5.1. Tested Pathogenic Bacteria

The antimicrobial activity was evaluated against a range of microorganisms isolated from food: Gram-positive bacteria, like *Staphylococcus aureus* subsp. *aureus* ATCC 25923, *Enterococcus faecalis* ATCC 29212 and *Listeria monocytogenes* ATCC 35152, and Gram-negative bacteria, such as *Salmonella enterica* subsp. *enterica* ATCC 14028, *Klebsiella pneumoniae* subsp. *pneumoniae* ATCC 13883 and *Escherichia coli* ATCC 25922. The bacterial viability was preserved by cultivating each pathogen on selective chromogenic substrates. These substrates were incubated in the dark at 37 °C for 24 h. Each bacterial strain was maintained at −80 °C.

Gram-positive and Gram-negative bacteria are the two main types of bacteria examined in this survey. These pathogens are representative of a wide range of disease models, primarily foodborne infections and human diseases (respiratory, urinary and gastrointestinal diseases).

#### 2.5.2. Evaluation of Antimicrobial Activity

The antimicrobial activity of aromatic plant extracts was determined using the agar dilution method [36]. Tubes (4) with 9 mL of an appropriate broth was filled with 4 distinct colonies—one of each microorganism—from a culture agar plate. The concentration of the bacterial suspension was modified to 1.0 × 10^8^ CFU/mL (0.5 McFarland). The above bacterial suspensions were blended with different concentrations of 4 aqueous plant extracts (40–3000 μg·mL^−1^), and 25 μL of this mixture were injected onto selective chromogenic substrates. The plates were kept in an incubation oven at 37 °C for 24 h and the bacterial growth was observed. MIC values were utilized to determine the antibacterial effectiveness of the samples [37].

### 2.6. Cell Culture and Viral Constructs

Antiviral activity was evaluated in Huh7.5 cells and Huh7-D2 cells. The latter cell line was generated using the DENV bicistronic replicon plasmid pD2-hRUPac, as described elsewhere [38]. Huh7-D2 cells harbor the subgenomic replicon of DENV serotype 2 (strain 16681) and simultaneously express the Renilla luciferase gene, the activity of which is indicative of viral replication. The cells were cultured in Dulbecco’s Modified Eagle Medium (DMEM) containing high glucose (4.5 g/L). The medium was supplemented with 0.1 mM non-essential amino acids, 2 mM L-glutamine, 100 U/mL penicillin, 100 µg/mL streptomycin and 10% (*v*/*v*) heat-inactivated fetal bovine serum (FBS), hereafter referred to as complete DMEM. Huh7-D2 cells were cultured under selective pressure with 0.25 µg/mL puromycin. All cell cultures were maintained at 37 °C in a humidified atmosphere with 5% (*v*/*v*) CO_2_. The plasmid containing the genomic sequence of the DENV-2 virus (strain 16,681), pFK-DVR2A (a reporter virus expressing Renilla luciferase), has previously been described in other studies [39].

### 2.7. Cytotoxicity Assay

The cytotoxicity of the aqueous plant extracts was evaluated in Huh7.5 cells using the AlamarBlue assay according to the manufacturer (ThermoFisher Scientific, Waltham, MA, USA). In brief, 10^4^ cells in 200 µL of complete DMEM per well were seeded in 96-well flat-bottom plates. Twenty-four hours post-seeding, the cells were treated with three concentrations of the extracts, determined on the basis of the maximum water uptake due to cellular osmosis limits (~10% *v*/*v*, 13.5 μg/mL), and incubated at 37 °C (5% CO_2_) for 48 h. Cells treated with only the diluent were used as the controls. After the 48-h incubation, the cells were supplemented with 44 μΜ (1X) AlamarBlue, followed by an additional 4-h incubation at 37 °C. Absorbance readings at 570 nm (reduced) and 600 nm (oxidized) were obtained using a plate reader. The concentration of the compound inducing 50% cell death (CC_50_) was determined by assessing the difference in dye reduction between control and treated cells (water-treated). Nonlinear regression analysis was used to determine the CC_50_ values following the conversion of drug concentrations to log-X format using Prism 6.0 software (GraphPad Software Inc., San Diego, CA, USA).

### 2.8. DENV Stocks and Cell Infection

The plasmid encoding the DENV genome (reporter variant) was linearized with XbaI prior to conducting in vitro transcription, as outlined in previous studies [40]. The integrity of the transcribed RNA was verified through electrophoresis on a denaturing agarose gel, while its concentration and quality were quantified using a Nanodrop spectrophotometer. The full-length DENV genome-derived RNAs generated by in vitro transcription were electroporated into VeroE6 cells [41]. Twenty-four hours after transfection, the medium was replaced with complete DMEM containing 15 mM HEPES (pH 7.5), and incubation was continued until the appearance of the DENV-induced cytopathic effect (CPE). Between days 4 and 7 post-transfection, the supernatants from the electroporated cells were harvested, pooled, filtered through a 0.45 μm filter, aliquoted and stored at −80 °C for future use as virus stock. A standard plaque assay was used to determine the virus titre following a 4-h inoculation of VeroE6 cells [42].

### 2.9. Cell-Based Antiviral Assays

Replicon assays were carried out in the Huh-D2 cell line by seeding 10^4^ cells in 200 µL of complete DMEM (supplemented with selection antibiotic) in a 96-well flat-bottom plate. Following 24 h of incubation at 37 °C (5% CO_2_), the culture medium was replaced with serial dilutions of the plant extracts (without puromycin) in complete DMEM, resulting in a final volume of 100 μL per well. The infection assays were conducted in Huh7.5 cells, and 24 h post-seeding, the medium was replaced, and the cells were infected with DENV stock (as described in Section 2.8) for 4 h. Subsequently, the medium was replaced with serial dilutions of the extracts, as previously mentioned. After a 2-day incubation at 37 °C, both replicon and infection assays were concluded by lysing the cells and measuring Renilla luciferase (R-Luc) activity. The relative luminescence units (RLU) were expressed as a percentage of the corresponding values from water-treated control cells. The compound concentration that caused a 50% reduction in the luciferase signal was defined as the half-maximal effective concentration (EC_50_). EC_50_ values were obtained via nonlinear regression analysis, with drug concentrations converted to logX utilizing Prism 5.0 software (GraphPad Software Inc.).

### 2.10. Luciferase Assay and Bradford Assays

Renilla luciferase (R-Luc) activity was assessed in cell lysates using 12 µM coelenterazine (Promega, Madison, WI, USA) in an assay buffer consisting of 50 mM potassium phosphate (pH 7.4), 500 mM NaCl and 1 mM EDTA. Measurements were obtained using a GloMax 20/20 single-tube luminometer (Promega Corporation, Madison, WI, USA) for a duration of 10 s. Luciferase activity was subsequently normalized to the total protein content, which was quantified using the Bradford assay reagent (Bio-Rad, Hercules, CA, USA).

### 2.11. Statistical Analysis

GraphPad Prism Software (version 6.0) was utilized in order to calculate the CC_50_ and EC_50_ values. Statistical analysis of the cytotoxicity values across the different extracts was performed using one-way ANOVA (using the maximal concentration of 10% *v*/*v*), followed by the appropriate post-hoc analysis (Tukey’s test). Comparisons of the cytotoxicity and antiviral activity values for each treatment condition versus mock-treated (control) cells were conducted with Student’s *t*-test in Microsoft Excel. Statistical significance was defined as a *p* value < 0.05.

## 3. Results and Discussion

### 3.1. Antioxidant Activity

Antioxidants are utilized to prevent and manage a range of human diseases, including cardiovascular diseases, Alzheimer’s disease, Parkinson’s disease, diabetes and cancer [43]. There are several in vitro techniques that can be used in order to evaluate the antioxidant properties of the extracts. One of the simplest and most used methods is the DPPH test. DPPH is a stable free radical with a deep purple colour generated by delocalization of electrons in all molecules [44]. The antioxidant’s ability to reduce the radical DPPH is demonstrated by the colour change of the radical from purple to yellow. This colour change is assessed by measuring the absorbance at 517 nm with the spectrophotometer [45]. The IC_50_ value was determined to evaluate the sample concentration necessary to reduce the radical by 50% [46].

In this research, the antioxidant activity of the standard compound gallic acid was estimated using the DPPH method (Figure S1). The equation below was derived from the standard reference curve:

(3) $$y=23.761ln\left(x\right)+3.897$$

The IC_50_ value of gallic acid was calculated as 6.96 ± 0.25 μg·mL^−1^.

**Figure 1:**
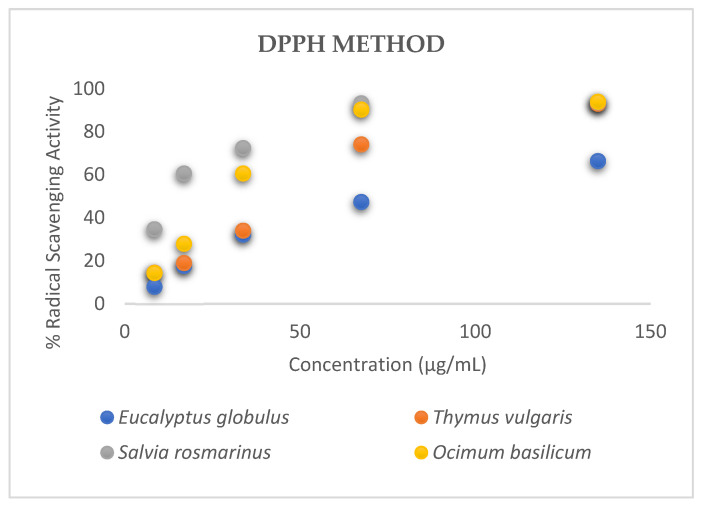
Capacity of the natural extracts to scavenge DPPH radicals.

**Figure 2:**
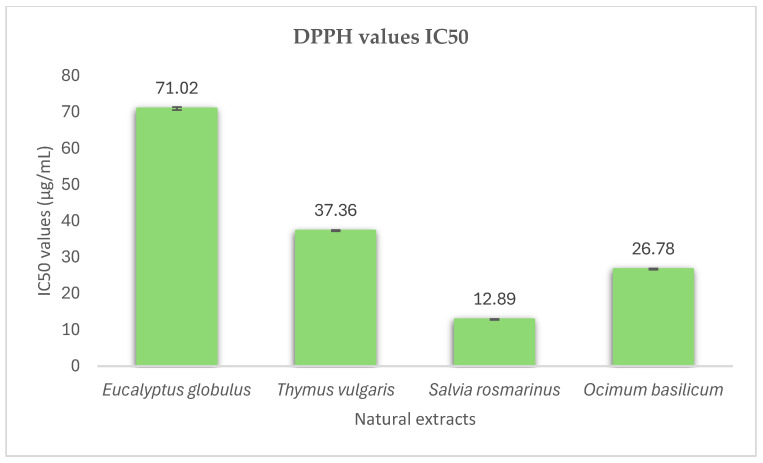
Comparison of antioxidant activity of natural extracts.

The IC_50_ values of the methanol extracts of the studied aromatic plants, *Eucalyptus globulus* L., *Thymus vulgaris* L., *Salvia rosmarinus* L. and *Ocimum basilicum* L. were 71.02 ± 0.42, 37.36 ± 0.15, 12.89 ± 0.11 and 26.78 ± 0.17 μg·mL^−1^, respectively (Figure 1 and Figure 2). A lower IC_50_ value reflects greater antioxidant activity in the samples. The extract of *Salvia rosmarinus* L. demonstrated the highest antioxidant activity among the other aromatic plants, as it had the lowest IC_50_ value compared to the other herbs (Figure 2). *Salvia rosmarinus* L. has high concentrations of bioactive compounds, especially phenolic acids (rosmarinic, benzoic, vanillic acid) [47]. These components and their synergistic action contribute to the effective antioxidant activity of this extract. It can be observed that all the examined plants have satisfactory antioxidant properties. These extracts are rich in polyphenols, like naringenin, luteolin, rutin, rosmarinic acid and quercetin. These phytochemical substances are responsible for the high antioxidant capacity of the samples [48].

The research conducted by Bencheikh et al. [49] revealed that the free radical scavenging ability of *Eucalyptus globulus* L. was greater than that found in our study, as the IC_50_ value was calculated as 18.9 ± 1.5 μg·mL^−1^. Mokhtari et al. [50] studied the extract of thyme and evaluated its antioxidant activity (IC_50_ = 69.39 ± 3.01 μg·mL^−1^). The extract of *Salvia rosmarinus* L. demonstrated high antioxidant potential (IC_50_ = 5.24 ± 0.12 μg·mL^−1^), according to previous studies [51]. The findings of this study are consistent with a scientific survey conducted by Naidu and colleagues [52] that examined the antioxidant potential of *Ocimum basilicum* L. (IC_50_ = 22 μg·mL^−1^). Consequently, the results of this research are comparable to those of other researchers.

### 3.2. Total Phenolic Content—Folin–Ciocalteu Method

The reductive potential of an antioxidant is determined using the electron transfer-based Folin–Ciocalteu method. This method has been extensively used to quantify the amount of polyphenols in the samples [53].

The phenol levels in the examined samples vary between 579.97 and 1532.84 μg GAE/mg DW. The highest total phenolic content was found in *Salvia rosmarinus* L. extracts with a value of 1532.84 ± 0.19 μg GAE/mg DW, whereas extracts of *Ocimum basilicum* L. had the lowest phenolic concentration (579.97 ± 0.28 μg GAE/mg DW). The quantity of phenolic compounds in the extracts of *Eucalyptus globulus* L. and *Thymus vulgaris* L. was 826.05 ± 0.84 and 628.14 ± 0.63 μg GAE/mg DW, respectively. The large amount of phenolic compounds is beneficial to human health. These substances defend against various diseases, like obesity, cardiovascular diseases, through their antioxidant properties as well as through the modulation of numerous cellular functions at multiple levels, such as protein phosphorylation and enzyme inhibition [54].

Stronger antioxidant activity is not always associated with increased phenolic content. An extract’s capacity to neutralize free radicals is not always strengthened by an increased phenol content. For example, the extract of *Eucalyptus globulus* L. had a satisfactory total phenolic content, while it had the lowest antioxidant potential among the others. This is due to the fact that the number and position of the hydroxyl groups and other substituents determine the antioxidant activity of the samples [55]. In addition, the synergistic effect of the phenols contributes to the effective antioxidant activity of the extracts.

The results of this study, regarding the number of phenolics, are contrasted with those of other research. Bencheikh et al. [49] found that the aqueous extract of *Eucalyptus globulus* L. had a total phenolic content of 280.63 ± 0.11 μg GAE/mg DW. Additionally, Abdul-Hafeez et al. [56] examined various medicinal plants and determined that the total phenolic content of *Thymus vulgaris* L. was 98.57 mg GAE/g DW. In a previous report, the total phenolic content of *Salvia rosmarinus* L. was measured to be 91 mg GAE/g DW [57]. According to the findings of another survey carried out by Naidu et al. [52], the amount of polyphenols in the extract of *Ocimum basilicum* L. was 45.38 ± 0.66 mg GAE/g DW. The soil, climate and geographical area are the main factors that significantly impact the price variations of phenolic compounds in the same plant species [58,59].

### 3.3. Antimicrobial Activity

Aromatic plant extracts, which have natural antimicrobial properties, are considered more secure than synthetic substances, more affordable, simple to use and they can provide significant therapeutic advantages [60]. Initially, the agar dilution method was used to assess the antibacterial activity of the four plant extracts against a range of microorganisms. These bacteria are commonly found in various illnesses and diseases. MIC values were used to estimate the antimicrobial effectiveness. MIC is the minimum concentration of the antimicrobial substance that prevents any discernible bacterial growth on the agar surface [61,62].

In this particular study, three Gram-positive, including *Staphylococcus aureus* subsp. *aureus* ATCC 25923, *Enterococcus faecalis* ATCC 29212 and *Listeria monocytogenes* ATCC 35152, and three Gram-negative bacteria, including *Salmonella enterica* subsp. *enterica* ATCC 14028, *Escherichia coli* ATCC 25922 and *Klebsiella pneumoniae* subsp. *pneumoniae* ATCC 13883, were used. The MIC values for the aqueous extract of *Eucalyptus globulus* L. ranged from 30 to 900 μg·mL^−1^. This extract was shown to inhibit Gram-positive bacterial growth at lower concentrations (30–135 μg·mL^−1^). Moreover, the extract of *Thymus vulgaris* L. had the most effective antibacterial activity against *Staphylococcus aureus* and *Listeria monocytogenes*, while it did not exhibit any antimicrobial effect against *Klebsiella pneumoniae*. The minimum inhibitory concentration values of the extract obtained from *Salvia rosmarinus* leaves were calculated to be 40–1000 μg·mL^−1^ and showed effective activity against all bacteria, while its ability to inhibit Gram-negative bacteria was weak (MIC ≥ 500 μg·mL^−1^). *Ocimum basilicum* L., which belongs to the Lamiaceae family, effectively inhibits the bacterial growth at a concentration range from 800 to 3000 μg·mL^−1^ (Table 1). It is observed that Gram-positive bacteria are more susceptible to the effects of the polyphenols included in the extracts, than Gram-negative pathogens. These results are consistent with the existing literature [57].

There are structural differences in the cell wall and membrane composition of Gram-positive bacteria that may explain their greater susceptibility to antimicrobial extracts. The exterior structure of Gram-positive bacteria is mostly composed of a thick, multilayered peptidoglycan cell wall (30–100 nm), while lacking an outer membrane [63,64,65]. As a result, antimicrobial agents can target that thick cell wall of the bacteria. On the other hand, Gram-negative bacteria have a thin peptidoglycan cell wall, which is enclosed by an outer membrane. The outer membrane is composed of lipopolysaccharides, which function as a protective barrier, preventing the entry of toxic substances into the bacteria [63]. Furthermore, a variety of proteins and enzymes found in the periplasmic space—the area between the inner and outer membranes—can neutralize or break down dangerous substances before they reach the bacterial cytoplasm or inner membrane [63]. Thus, the absence of an outer membrane, the less complex and more accessible cell wall structure, and the ease with which antimicrobial agents can target the exposed peptidoglycan layers of Gram-positive bacteria are the main causes of their increased vulnerability to antimicrobial extracts. However, the exact mechanisms of antimicrobials are unclear, and further experiments are necessary.

### 3.4. Cytotoxicity Assay

The cytotoxic effects of the plant extracts on Huh 7.5 cells were assessed using the Alamar Blue assay, a widely recognised technique for measuring cell viability. This assay relies on the capacity of viable cells to convert resazurin (the primary component of Alamar Blue) into a fluorescent product, which can be quantified [66].

**Figure 3:**
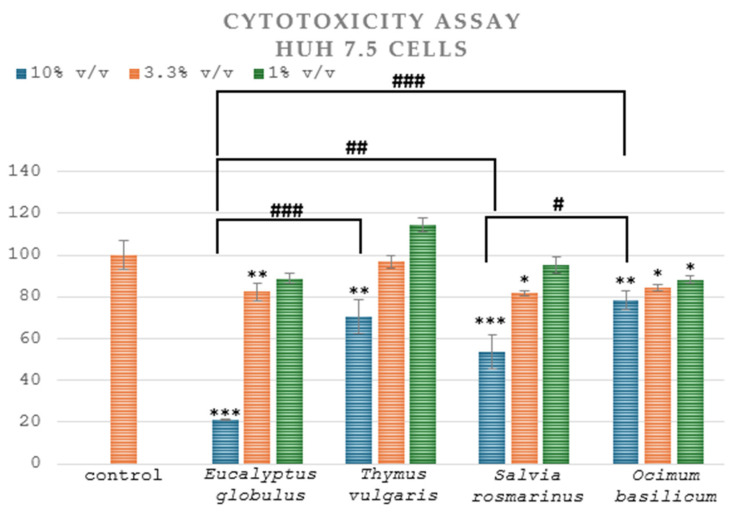
Cytotoxicity of the plant extracts. * *p* < 0.05, ** *p* < 0.01 and *** *p* < 0.001 vs. control (Student’s *t*-test). # *p* < 0.05, ## *p* < 0.01 and ### *p* < 0.001 across extracts (one-way ANOVA).

In this study, the plant extracts demonstrated different levels of cytotoxicity against Huh7.5 cells. Huh7 liver cell lines were applied as a model for human normal hepatocytes and as a novel approach to imitate the liver environment. Huh7.5 cells were treated for 48 h with the indicated concentrations of the plant extracts or were mock-treated (control) with the solvent. Cell viability was determined by comparing it to control cells, which exhibited 100% viability and have were not treated. Table 2 displays the sample concentrations required to decrease 50% of cell growth. The extract of *Eucalyptus globulus* L. that exhibited a lower CC_50_ value had the highest cytotoxicity. The results indicated a dose-dependent effect, where higher concentrations of the plant extracts caused a greater reduction in cell viability. The samples were examined at three different concentrations: 10% *v*/*v*, 3.3% *v*/*v* and 1% *v*/*v*. Plant extracts at a 10% *v*/*v* concentration led to a considerably greater inhibition of cell proliferation in comparison to control (Figure 3). On the other hand, treatment of cells with a 1% *v*/*v* concentration of plant extract did not significantly impact the viability of Huh7.5 cells (Figure 3). The effective action of plant extracts against hepatocellular carcinoma cells is due to their rich content in bioactive compounds, such as flavonoids, alkaloids or phenolic compounds [67].

Polyphenols have been extensively researched for their capacity to regulate a number of biological functions, including oxidative stress, apoptosis and cell proliferation [68]. Research shows that polyphenols can prevent the progression of liver cancer and preserve normal liver function by affecting metabolic pathways. Numerous studies have shown that a range of polyphenols has anti-hepatocarcinoma and liver-protective properties. Their mechanisms are also varied, impacting the expression of certain proteins or genes to suppress tumour cell proliferation, autophagy, apoptosis, metastasis and metabolic processes [69,70]. Teixeira et al. [71] studied different cell lines and underlined the anticancer effects of the extract of *Eucalyptus globulus* leaves on colorectal (HCT-15), pancreatic (PANC-1) and non-small cell lung cancer (NCI-H460). Moreover, the extract of *Thymus vulgaris* L., in addition to its effective action against liver diseases, exhibits anticancer properties against two leukaemia cell lines (CCRF-CEM and CEM/ADR5000) and different myeloma cell lines [72]. *Salvia rosmarinus* L. extracts contain bioactive compounds that have beneficial effects on cell lines, such as human breast adenocarcinoma cell lines (MCF-7), cervical carcinoma cell lines (HeLa) and hepatocellular carcinoma cell lines [73]. According to Palanichamy et al. [74], extracts of *Ocimum basilicum* L. had also antiproliferative properties against A549 (lung cancer), MCF-7 (breast cancer) and Huh7 cell lines.

Huh7.5 cells were treated for 48 h with the indicated concentrations of the plant extracts or were mock-treated (control) with the solvent. Following treatment, cell viability was measured using the AlamarBlue assay. The bars represent the mean percentage of viable cells compared to the control, while the error bars correspond to the standard deviation of values obtained from three independent experiments, each performed in triplicate (Figure 3). The half-maximal cytotoxic concentration (CC_50_) was calculated based on the concentration of extracts required to reduce cell viability by 50%, using Prism 6.0 software (GraphPad Software Inc.), and the results are presented in Table 2.

*Eucalyptus globulus* L. exhibited the highest cytotoxicity with a CC_50_ of 5.94% *v*/*v* ± 0.04, compared to the other extracts, which had CC_50_ values > 10% *v*/*v*. One-way ANOVA analysis across extracts using the highest concentration tested (10% *v*/*v*) showed that *Ocimum basilicum* L. and *Thymus vulgaris* L. were the safest, followed by *Salvia rosmarinus* L. and *Eucalyptus globulus* L.

### 3.5. Cell-Based Antivirus Assays

#### Plant Extracts Positively Affect DENV Replication and Attenuate the Infection

The various clinical manifestations of dengue virus pose a significant public health concern, and there is no specific and efficient antiviral treatment. Supportive care is the sole treatment for combating this virus [75]. Another challenge in the quest for antiviral substances is their variable selectivity towards various types of viral strains [76]. Based on this information, experiments focusing on the efficacy of various herbal products against viruses would benefit the scientific community.

As is widely known, plant extracts are rich in phenolic compounds. These substances have many clinical applications, and they are considered antiviral agents suitable for humans. This property may be due to their chemical structure, as they contain aromatic rings linked to at least one or more hydroxyl substituents [77]. For example, polyphenols, included in the extract of *Glycyrrhiza uralensis*, can combat rotavirus. It has also been demonstrated that curcumin prevents hepatitis B virus, and resveratrol inhibits varicella-zoster virus [78]. Furthermore, previous studies have highlighted that plant extracts can exhibit antiviral protection against DENV as well as HCV viruses due to their rich content of bioactive molecules that can act on multiple stages of the viral replication cycle [79,80,81,82,83].

**Figure 4:**
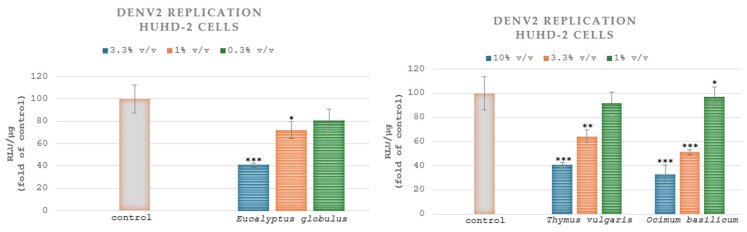
Effect of plant extracts on DENV serotype 2 replication. Values from control cells were set to one hundred. Bars represent mean values from three independent experiments in triplicate. Error bars indicate standard deviations. * *p* < 0.05, ** *p* < 0.01 and *** *p* < 0.001 vs. control (Student’s *t*-test).

In HuhD-2 cell cultures infected with the DENV serotype 2 (strain 16681) and treated with the extracts of *Eucalyptus globulus* L., *Thymus vulgaris* L., *Salvia rosmarinus* L. and *Ocimum basilicum* L., the EC_50_ values were estimated at 2.37% *v*/*v* ± 0.6, 6.60% *v*/*v* ± 0.96, >10% *v*/*v* and 3.97% *v*/*v* ± 0.36, respectively. Thus, the aqueous extract of *Eucalyptus globulus* L. demonstrated the greatest inhibitory effects on DENV-2 replication. The HuhD-2 cells were exposed to three different concentrations of the samples. A statistically significant reduction was observed in comparison to the control group (cells without extract) when the cells were treated with the aqueous extracts of *Eucalyptus globulus* L. (1% *v*/*v*, 3.3% *v*/*v*), *Thymus vulgaris* L. (3.3% *v*/*v*, 10% *v*/*v*) and *Ocimum basilicum* L. (1% *v*/*v*, 3.3% *v*/*v* and 10% *v*/*v*) (Figure 4). The aqueous extract of *Eucalyptus globulus* L. demonstrated the greatest inhibitory effects on DENV-2 replication, producing a 59% reduction at its maximum tested concentration of 3.3% *v*/*v,* followed by a 27.7% and 19% reduction at concentrations of 1% *v*/*v* and 0.3% *v*/*v,* respectively (Figure 4).

**Figure 5:**
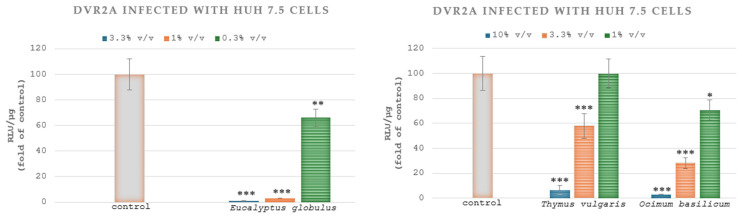
Effect of plant extracts on DENV infection. Values from control cells were set to one hundred. Bars represent mean values from three independent experiments in triplicate. Error bars indicate standard deviations. * *p* < 0.05, ** *p* < 0.01 and *** *p* < 0.001 vs. control (Student’s *t*-test).

In Huh7.5 cell cultures infected with DVR2A and treated with the extracts of *Eucalyptus globulus* L., *Thymus vulgaris* L., *Salvia rosmarinus* L. and *Ocimum basilicum* L., the EC_50_ values were calculated at 0.36% *v*/*v* ± 0.004, 3.72% *v*/*v* ± 0.13 and >10% *v*/*v* and 1.76% *v*/*v* ± 0.11, respectively. Therefore, the aqueous extract of *Eucalyptus globulus* L. exhibited the highest inhibitory effects on DVR2A. Figure 5 displays the impact of plant extracts on DVR2A. Specifically, the Huh7.5 cells were treated with three different concentrations of the plants. A statistically significant decrease was noted when the cells were treated with the aqueous extract of *Eucalyptus globulus* L. (0.3% *v*/*v*, 1% *v*/*v*, 3.3% *v*/*v*), *Thymus vulgaris* L. (3.3% *v*/*v*, 10% *v*/*v*) and *Ocimum basilicum* L. (1% *v*/*v*, 3.3% *v*/*v*, 10% *v*/*v*) compared to the control group. Figure 4 and Figure 5 do not illustrate the effect of *Salvia rosmarinus* L. on DENV-2 and DVRA2A, respectively, because this extract exhibited 100% RLU/μg in the highest concentration used for the treatment.

HuhD-2 cells, harbouring the DENV serotype 2 (strain 16681) subgenomic replicon, were treated for 48 h with the indicated concentrations of the plant extracts (10, 3.3 and 1% *v*/*v* for *Thymus vulgaris* L. and *Ocimum basilicum* L., and 3.3, 1 and 0.3% *v*/*v* for *Eucalyptus globulus* L.) or were mock-treated (control) with the solvent. Viral RNA replication-derived Renilla luciferase activity values were determined and subsequently normalized to the total protein amount, expressed as RLU/µg of total protein (Figure 4). The median effective concentration (EC_50_) of the compounds, reducing luciferase signal by 50%, was determined by nonlinear regression analysis using Prism 6.0 software (GraphPad Software Inc.).

Huh7.5 cells were exposed to DENV (MOI = 0.05) for 4 h. Then, the cells were further cultured for 72 h in a culture medium containing the extracts in the serial concentrations shown above (10, 3.3 and 1% *v*/*v* for *Thymus vulgaris* L. and *Ocimum basilicum* L., and 3.3, 1 and 0.3% *v*/*v* for *Eucalyptus globulus* L.) or were mock-treated (control) with the solvent. Renilla luciferase activity, resulting from viral RNA replication, was quantified and normalized relative to the total protein content, expressed as RLU/µg of total protein (Figure 5). The median effective concentration (EC_50_) of the compounds, reducing luciferase signal by 50%, was determined by nonlinear regression analysis using Prism 6.0 software (GraphPad Software Inc.).

To the best of our knowledge, this is the first time that *Eucalyptus globulus* L., *Thymus vulgaris* L. and *Ocimum basilicum* L. exhibited promising antiviral activity against the dengue virus. However, the antiviral activity of *Ocimum basilicum* L. against dengue virus (DENV) and chikungunya virus (CHIKV) has been shown before [84].

In this study, we employed a subgenomic replicon of DENV serotype 2, which enables us to study a specific aspect of the viral life cycle, namely the genome replication stage. The replicon co-expresses the Renilla luciferase reference gene, the activity of which serves as an indicator of viral replication efficacy. The effect of the extracts observed in this system strongly suggests that the mechanism of action involves, at least in part, inhibition of the viral genome replication machinery. Additionally, the more pronounced antiviral activity observed in the context of the full-length virus, as indicated by the lower EC_50_ values, compared to the ones derived from the subgenomic replicon, supports the possibility that the compounds also target other stages of the viral life cycle. The impact of specific bioactive compounds isolated from each of the three plants has been previously confirmed to inhibit dengue virus genome replication through interaction with the virus non-structural proteins, providing further evidence to support the aforementioned claim. Specifically, among the compounds shown to have anti-dengue activity, the flavonoids naringenin and eriodyctiol have been identified in all three plants studied [85,86,87,88]. Naringenin has been reported to impair DENV-2 replication primarily by interacting with NS5-methyltransferase and by weakly binding to the NS3 helicase–protease domains [89,90]. Similarly, eriodictyol has been shown to bind to the NS3 protease and interfere with the protease-activating NS2B protein [91]. Another important flavonoid, quercetin, identified in *Thymus vulgaris* L. [92], inhibits DENV-2 and DENV-3 replication through multiple mechanisms. It binds to the NS2B–NS3 protease, thereby interfering with polyprotein processing [93], and it also prevents phosphorylation of the NS3 protein, a key step in viral RNA replication [94]. In addition, p-coumaric acid, luteolin and rosmarinic acid, which are contained in both *Eucalyptus globulus* L. and *Thymus vulgaris* L. [85,92], have antiviral effects. P-coumaric acid impedes DENV-2 replication by binding to the NS2B–NS3 protease [95]. Luteolin hinders the later stages of the dengue virus life cycle by inhibiting furin, a host cell protease involved in viral maturation [96], and rosmarinic acid binds to the envelope domain III (EDIII) protein of all four DENV serotypes, thereby preventing viral entry into host cells [97]. It also neutralizes all four serotypes, as confirmed through plaque assays [98].

## 4. Conclusions

There has been a significant global growth in the assessment of medicinal plants for their biological activity. This demonstrates that the compounds extracted from aromatic plants are undoubtedly valuable for use in alternative medicine.

The DPPH method was conducted to evaluate the four extracts’ ability to neutralize free radicals. All of the examined extracts demonstrated antioxidant capacity, with *Salvia rosmarinus* L. and *Ocimum basilicum* L. being the most effective, as they have lower IC_50_ values. Furthermore, the findings suggest that the studied natural extracts exhibited antimicrobial action against both Gram-positive and Gram-negative pathogenic bacteria. Gram-negative bacteria are more resistant to the extracts than Gram-positive pathogens. This might be explained by differences in the cell membranes of bacteria. Therefore, a novel strategy to counter the rising problem of antimicrobial resistance is the utilization of natural herbs.

The cytotoxicity assay was assessed with the AlamarBlue method, and plant extracts, especially *Ocimum basilicum* L. and *Thymus vulgaris* L., demonstrated low cytotoxicity against human hepatoma cells. The plants’ high polyphenol content is one of the reasons for their potent anticytotoxic and, consequently, anticancer effects. However, the effectiveness of a plant extract in fighting cancer is a complex issue and is determined by a range of factors. Although some plant extracts are effective as antioxidants and antimicrobials agents, this does not guarantee that they will always have a positive impact on cancer treatment or prevention. In addition, our findings suggest that *Eucalyptus globulus* L., *Thymus vulgaris* L., *Salvia rosmarinus* L. and *Ocimum basilicum* L. can inhibit DENV infection and might act as potent therapeutic agents.

The results of this study have great potential for the development of new antiviral and antimicrobial treatments. Natural substances may have a number of benefits over manufactured medications, including being more readily available, biodegradable and having fewer adverse effects. The development of plant-based antiviral drugs offers a substitute for conventional antiviral medications. Plant-based treatments might play a key role in public health campaigns in areas where access to synthetic medications is restricted. Furthermore, the development of plant-based antimicrobial drugs is one potential solution to the rising issue of antibiotic resistance. The phytochemical substances can contribute to the creation of novel antibiotics. It would also be interesting to examine the creation of hybrid molecules, which combine synthetic drugs with substances extracted from plants. This combination may result in powerful therapies with a wide range of antiviral and antibacterial activities. However, these applications of plants appear to have some limitations. First, there is a lack of in vivo validation; therefore the effectiveness and safety of the natural components are controversial. These studies are necessary in order to determine whether plant extracts are equally effective in humans as in laboratories. In addition, the variability in extract composition can cause problems with quality control and repeatability. Thus, it is challenging to standardize plant-based drugs for therapeutic applications. Numerous variables, including geographic location, plant growth conditions and harvesting practices can affect the composition of natural extracts. The potential toxicity of plant-based drugs is another limitation. Despite being natural, plants can contain substances that become toxic at high doses. Clinical trials and toxicological research are required to evaluate the safety of these substances. The limitations must be thoroughly examined via scientific research in order to confirm that natural extracts can be instrumental in the treatment of infectious diseases.

In conclusion, this study underscores the potential therapeutic uses of phytochemical substances, focusing on the antioxidant, antibacterial, cytotoxic and antiviral properties. The results indicate that the studied extracts are a viable source of natural treatments. The findings of this survey can serve as a reference point for future research on plant species. It would be interesting to focus on the isolation and quantification of bioactive compounds (flavonoids, terpenoids, phenolic acids), using advanced chromatography methods. Furthermore, research on the toxicity, synergistic action and bioavailability of the phenolic substances both in vitro and in vivo will be essential for scientists to develop therapies with fewer adverse effects. These discoveries will have a very significant impact on the health sector.

## Figures and Tables

**Table 1:** Antibacterial activity of four natural extracts and their MIC values.

	MIC (Minimum Inhibitory Concentration) μg·mL^−1^					
Aqueοus Νatural Extracts	*S. aureus*	*E. faecalis*	*L. monocytogenes*	*S. enterica*	*E. coli*	*K. pneumoniae*
*Eucalyptus globulus* L.	135	120	30	120	135	900
*Thymus vulgaris* L.	135	200	135	1000	1000	nd
*Salvia rosmarinus* L.	40	80	40	1000	1000	500
*Ocimum basilicum* L.	800	800	1000	1000	3000	3000

**Table 2:** Determination of the CC_50_ values of the four medicinal aqueous plant extracts.

Plant Extract	CC_50_ (% *v*/*v*)
*Eucalyptus globulus* L.	5.94 ± 0.04
*Thymus vulgaris* L.	>10
*Salvia rosmarinus* L.	>10
*Ocimum basilicum* L.	>10

## Data Availability

The original contributions presented in this study are included in the article/Supplementary Materials. Further inquiries can be directed to the corresponding author.

## Supplementary Materials

The following supporting information can be downloaded at: https://www.mdpi.com/article/10.3390/microorganisms13010177/s1, Figure S1: Reference curve of the standard gallic acid after measuring its antioxidant activity.
