# Supplementary material for: In Vitro Determination of Antimicrobial, Antioxidant and Antiviral Properties of Greek Plant Extracts
Source: Microorganisms. 2025 Jan 16;13(1):177. doi: 10.3390/microorganisms13010177 (PMC11767790; doi:10.3390/microorganisms13010177)
Supplement: Supplementary file 1 [file microorganisms-13-00177-s001.zip › microorganisms-3418611-supplementary.pdf]

microorganisms

Supplementary Materials

In Vitro Determination of Antimicrobial, Antioxidant and Antiviral Properties of Greek Plant Extracts

Aliki Tsakni [1], Eirini Kyriakopoulou [2], Sophia Letsiou [1,3], Panagiotis Halvatsiotis [4], Haralambos Rigopoulos [1], Niki Vassilaki [2] and Dimitra Houhoula [1,*]

1 Department of Food Science and Technology, Faculty of Food Sciences, University of West Attica, 12243 Athens, Greece
2 Laboratory of Molecular Virology, Hellenic Pasteur Institute, 11521 Athens, Greece
3 Department of Biomedical Science, University of West Attica, 12243 Athens, Greece
4 2nd Propaedeutic Department of Internal Medicine, Medical School, National and Kapodistrian University of Athens, "ATTIKON" University Hospital, 12461 Chaidari, Greece
* Correspondence: dhouhoula@uniwa.gr

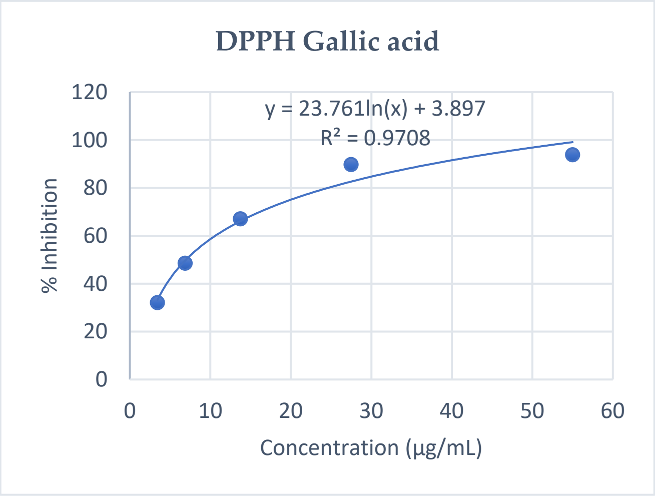

Figure S1. Reference curve of the standard gallic acid after measuring its antioxidant activity.
